# Co-Formulation of Edamame-Based Beverage with Coconut Derivatives Enhances Nutritional Quality, Antioxidant Capacity, Flavor Profile, and Physical Stability

**DOI:** 10.3390/foods14193321

**Published:** 2025-09-25

**Authors:** Phatthranit Klinmalai, Khwanchat Promhuad, Atcharawan Srisa, Aiyaporn Sathawarintu, Nathdanai Harnkarnsujarit

**Affiliations:** 1 Faculty of Agro-Industry, Chiang Mai University, Samut Sakhon 74000, Thailand; phatthranit.k@cmu.ac.th (P.K.); aiyaporn_s@cmu.ac.th (A.S.); 2 Department of Packaging and Materials Technology, Faculty of Agro-Industry, Kasetsart University, Bangkok 10900, Thailand; khwanchatpromhuad@gmail.com (K.P.); atcharawan.sri@ku.th (A.S.)

**Keywords:** edamame-based beverage, coconut, functional beverage, antioxidant, amino acid, plant based

## Abstract

Edamame beans, rich in protein, essential amino acids, and antioxidant compounds, are promising substrates for novel plant-based beverages. This study developed and comprehensively characterized edamame-based beverage formulations with enhanced nutritional and functional attributes. Six formulations were prepared at edamame–water ratios of 1:3 or 1:6, incorporating either coconut water or coconut milk. Physicochemical analyses included particle size distribution, viscosity, amino acid and mineral profiles, antioxidant activity, volatile compounds, and storage stability. Nutritional analysis revealed that the ECM (1:3) formulation exhibited the highest protein content (3.68 g/100 g), while all formulations delivered essential minerals, with calcium levels ranging from 19.25% to 27.64% of total mineral content. ECW formulations were particularly rich in potassium, calcium, and phosphorus, whereas the pure edamame-based beverage had higher concentrations of sulfur and magnesium. The E (1:3) formulation demonstrated the highest total amino acid concentration (24.85 mg/mL), with glutamic and aspartic acids predominating compounds known to contribute to umami taste and buffering capacity. Higher edamame concentrations also resulted in significantly greater total phenolic (16.25 mg GAE/100 mL) and flavonoid content (6.42 mg QE/100 mL), which correlated with improved DPPH radical scavenging activity. The addition of coconut milk significantly reduced particle size, improved emulsion stability, and increased viscosity, while also masking undesirable volatile compounds such as hexanal, commonly associated with the beany aroma of legumes. These findings highlight the synergistic potential of blending edamame with coconut-based ingredients to produce nutrient-dense, sensorially acceptable, and shelf-stable plant-based beverages.

## 1. Introduction

In recent years, the global demand for plant-based beverage alternatives has increased significantly, driven by heightened consumer awareness of health benefits, environmental sustainability, and ethical concerns associated with traditional dairy production [1,2,3]. These alternative beverages offer a more sustainable option due to their lower environmental footprint when compared to animal-based milk products [4]. Additionally, the rising incidence of lactose intolerance and cow’s milk protein allergy among global populations further contributes to the shift away from conventional dairy consumption [2,5,6]. Plant-based beverages derived from soybeans, oats, almonds, legumes, and other botanical sources have garnered increasing consumer acceptance due to their nutritional and functional benefits. These beverages are naturally devoid of cholesterol and bovine hormones and are often rich in bioactive compounds such as phytosterols, isoflavonoids, polyphenols, unsaturated fatty acids, and dietary fiber [2,7]. Among these sources, edamame has emerged as a particularly promising raw material for milk alternatives due to its high-quality protein content, low glycemic index, and abundance of dietary fiber and essential amino acids [8,9].

Edamame contains a favorable amino acid profile, including leucine and arginine, which have been shown to stimulate insulin secretion and may support glycemic regulation in diabetic individuals [10,11]. Additionally, it is a good source of unsaturated fatty acids (notably linoleic and linolenic acids), saponins, and isoflavones, all of which contribute to its potential health benefits, such as antioxidant activity, anti-inflammatory effects, and cardiovascular protection [11,12]. Given these characteristics, the edamame-based beverage has potential applications as a functional beverage for individuals with metabolic disorders, including type 2 diabetes and chronic kidney disease (CKD), where dietary interventions play a critical role in health management [13]. Despite these benefits, the edamame-based beverage faces several formulation challenges. Its colloidal nature and high protein and fiber content predispose it to phase separation, sedimentation, and a short shelf life. These physical instabilities are influenced by key parameters such as particle size distribution, viscosity, and processing techniques [14]. Pasteurization is commonly employed to improve microbial safety; however, thermal treatment can also impact nutritional and bioactive components, necessitating careful optimization [15]. Moreover, edamame-based beverages are often characterized by a pronounced “beany” flavor, primarily due to volatile aldehydes (e.g., hexanal and pentanal) and alcohols formed by lipoxygenase-mediated oxidation of unsaturated lipids [16]. Addressing these sensory limitations remains a priority for enhancing product acceptability. Coconut-based ingredients, including coconut milk and coconut water, present valuable opportunities to improve both the sensory profile and functional characteristics of plant-based beverage formulations. Coconut milk provides a creamy texture and tropical aroma due to its high fat and mineral content [2], whereas coconut water contributes a refreshing taste and essential electrolytes such as potassium and magnesium [17,18]. While coconut milk may increase viscosity and improve mouthfeel, its high saturated fat content can pose nutritional concerns [5,19]. On the other hand, coconut water is low in fat and calories but rich in antioxidants and minerals, offering potential functional benefits. Although the individual health benefits and physicochemical properties of both edamame and coconut-based products have been previously documented, little attention has been given to their combined use. Blending edamame with coconut milk or water may not only mitigate the undesirable sensory traits but also enhance the overall nutritional profile and physicochemical stability of the final product. However, systematic investigation of such blends, particularly in terms of their impact on particle size distribution, rheological behavior, antioxidant activity, and bioactive compound content, is currently lacking.

Therefore, the objective of this study is to evaluate the impact of coconut milk and coconut water on the physicochemical, nutritional, and functional properties of edamame-based plant beverage. Specifically, the study investigates particle size distribution, viscosity, separation index, antioxidant activity, total phenolic and flavonoid content, amino acid profile, mineral composition, and volatile compounds. The findings are expected to contribute valuable insights for the development of novel, functional, and consumer-acceptable plant-based beverages.

## 2. Materials and Methods

### 2.1. Preparation of Edamame-Based Beverage

Frozen edamame beans (*Glycine max* (L.) Merr.), sourced from northern Thailand, were purchased from Food Project (Siam) Co., Ltd. (Bangkok, Thailand). Coconut milk (CM) and coconut water (CW) were obtained from Theppadungporn Coconut Co., Ltd. (Chaokoh factory) (Nakhon Pathom, Thailand). According to the manufacturer’s specification, CM contained 24 g fat/100 g, 2.3 g protein/100 g, 6 g carbohydrate/100 g, and 263 mg potassium/100 g, whereas CW contained 0 g fat/100 g, 0 g protein/100 g, 14.55 g carbohydrate/100 g, and 545.45 mg potassium/100 g. Edamame-based beverage preparation followed a modified method adapted from da Silveira Maia [13]. Initially, the edamame beans were boiled at 100 °C for 15 min, then strained and cooled to room temperature. Subsequently, the boiled beans were blended with water at weight ratios of 1:3 and 1:6 (denoted as E (1:3) and E (1:6), respectively) using a 1600-watt blender (BL91HD65, high-speed blender, Tefal, Thailand) for 3 min. The resulting mixture was then filtered through cheesecloth to remove insoluble residues. In alternative formulations, either coconut milk (CM) or coconut water (CW) was used to replace half of the water content in the blending process. Accordingly, the ECW (1:3) and ECW (1:6) samples were prepared by blending boiled edamame, water, and coconut water at ratios of 1:1.5:1.5 and 1:3:3, respectively. Similarly, the ECM (1:3) and ECM (1:6) formulations involved blending boiled edamame with water and coconut milk at the same respective ratios of 1:1.5:1.5 and 1:3:3. All formulations followed the same production steps as described above. Finally, each edamame-based beverage sample was pasteurized by heating to 72 °C for 15 s and stored at 4 °C until further analysis.

### 2.2. Proximate Composition

Proximate composition (moisture, crude protein, crude fat, total ash, and crude fiber) was determined according to AOAC (2000) [20]. Analyses were performed in triplicate. Moisture was determined gravimetrically by drying samples (2 g) at 105 °C until constant weight. Crude protein content was analyzed via the Kjeldahl method, converting total nitrogen (from a 2 g sample) to protein content using a factor of 6.25. Crude fat was quantified using Soxhlet extraction (petroleum ether, boiling range 40–60 °C) from 5 g samples. Ash content was determined by incineration (550 °C for 5 h), and crude fiber was measured by H_2_SO_4_ and NaOH digestion followed by incineration at 550 °C for 5 h.

### 2.3. Mineral Composition

Mineral composition was analyzed using a Wavelength Dispersive X-ray Fluorescence Spectrometer (WD-XRF, ZSX Primus IV, Rigaku, Tokyo, Japan) operated in qualitative mode with a Rh X-ray tube. Samples were prepared by two methods following Moriyama (2017): (i) liquid cell method, where ~20 mL liquid was loaded into a polypropylene-film cell (20 mm mask), and (ii) droplet method, where 300 µL was pipetted onto filter paper MicroCarry, vacuum-dried at 30 °C for 8 h, and analyzed as a solid sample (10 mm mask). Each sample was tested in triplicate. Results are reported as semi-quantitative relative concentrations (mass%), not absolute quantitative values.

### 2.4. Amino Acid Profile

Protein hydrolysis was performed using 6 M hydrochloric acid (HCl) at 110 °C for 24 h in vacuum-sealed glass tubes to prevent oxidation. Approximately 10–20 mg of protein equivalent from the freeze-dried sample was hydrolyzed in the presence of 0.1% phenol (to protect tyrosine residues). For sulfur-containing amino acids (e.g., cysteine, methionine), performic acid oxidation was used prior to acid hydrolysis, as recommended by Waters and standard protocols. After hydrolysis, samples were neutralized, dried under vacuum, and redissolved in borate buffer. Derivatization was performed using the AccQ-Tag™ reagent kit (Waters corporation, Milford, MA, USA), which contains 6-aminoquinolyl-N-hydroxysuccinimidyl carbamate (AQC) as the derivatizing agent. Specifically, 10 µL of sample hydrolysate was mixed with 70 µL of AccQ-Tag borate buffer and 20 µL of AccQ-Tag reagent solution, followed by vortexing and incubation at 55 °C for 10 min to ensure complete derivatization. Amino acid derivatives were separated using reverse-phase HPLC with a Waters AccQ-Tag™ Ultra column (2.1 × 100 mm, 1.7 µm particle size) maintained at 37 °C. The elution was performed using a binary gradient system of AccQ-Tag eluents A and B, following the manufacturer’s gradient program. Detection was conducted with a fluorescence detector set at excitation 250 nm and emission 395 nm. Chromatograms were analyzed using Empower™ 3.8.1 software (Waters corporation, Milford, MA, USA) [21]. The amino acid concentrations are expressed in mg/mL, with values normalized to protein mass.

### 2.5. Preparation of Hydroalcoholic Extracts

The antioxidant activity, total phenolic content, and flavonoid content of the beverage samples were measured by solvent extraction with the method of Moretto [12]. A 5 mL of edamame-based beverage was mixed with 25 mL of 80% ethanol. The mixture was then sonicated for 20 min while maintaining temperature control by adding ice during the extraction process. After sonication, the sample was centrifuged at 5000 rpm for 20 min at 4 °C. The supernatant was filtered using Whatman No. 1 filter paper, yielding the edamame-based beverage extract. This extract was subsequently used for the analysis of antioxidant activity, total phenolic content, and flavonoid content.

### 2.6. Total Phenolic Content

Total phenolics were measured following Taesuk [7]. An aliquot of 0.1 mL of the extract was mixed with 0.9 milliliters of distilled water, followed by the addition of 0.1 mL of Folin–Ciocalteu reagent. The mixture was blended and left to stand for 6 min. Then, 1 mL of 7% Na_2_CO_3_ solution was added and mixed thoroughly. The mixture was left at room temperature for 90 min. Absorbance was then measured at a wavelength of 760 nm. Each sample was analyzed in triplicate, and results are expressed as mL of gallic acid equivalents (GAE) per 100 mL of edamame-based beverage.

### 2.7. Total Flavonoid Content

Total flavonoid content was measured following the method of Taesuk [7], with slight modification. An aliquot of 0.25 mL of the extract was mixed with 0.075 mL of 5% NaNO_2_ solution and left to stand at room temperature for 6 min. Then, 0.15 mL of 10% AlCl_3_ solution was added, mixed thoroughly, and left to stand for another 5 min at room temperature. Next, 5 milliliters of 1 M NaOH, followed by 2.5 mL of distilled water, were added, and the mixture was homogenized. The absorbance was measured at a wavelength of 510 nm. Each sample was analyzed in triplicate, and the results are expressed as milliliters of quercetin equivalents (QE) per 100 mL of edamame-based beverage.

### 2.8. Antioxidant Activity

Antioxidant activity was measured following the method of Taesuk [7] with slight modification. An aliquot of 0.05 mL of the extract was mixed with 2 mL of 25 ppm DPPH solution. The mixture was blended thoroughly and left to stand in the dark at room temperature for 30 min. The absorbance was then measured at a wavelength of 517 nm using a spectrophotometer (Genesys 20 model). Each sample was analyzed in triplicate, and the results are expressed as the percentage of DPPH radical scavenging activity.

### 2.9. Particle Size and Distribution

The particle size of the samples was measured using a laser light scattering particle size analyzer (Mastersizer 3000; Malvern, Worcestershire, UK) under wet dispersion conditions (Hydro EV). Prior to analysis, the samples were shaken by inverting the container 10 times to ensure homogeneity, as phase separation had occurred. The analysis was conducted at 25 °C using refractive index values of 1.52 and 1.33 [14].

### 2.10. Rheological Behavior

The rheological behavior of the liquid samples was measured using a viscometer (Series RV Model DV-III, Middleboro, MA, USA) equipped with a UL adapter. A sample volume of 16 mL was used for each analysis, conducted at room temperature with a maximum rotational speed of 80 revolutions per min (rpm) and shear stress (Pa).

### 2.11. Separation Index

The separation index (SI) of edamame-based beverage samples was evaluated following the method described by Ren [22]. The edamame-based beverage was stored in glass bottles at 4 °C with 80% relative humidity. The SI of samples stored at 4 °C, 80% RH, was monitored on days 0, 4, 8, 12, 16, and 20, and calculated using the following equation:

(1) SI = (1 − Hu/Ht) × 100%

where Ht is the total height of the sample in the bottle (mm), and Hu is the height of the upper separated layer (mm).

### 2.12. Phenolic Compounds

HPLC analysis (Agilent 1100 series, diode array detection, Phenomenex LUNA C18 column) identified phenolic compounds according to the modified method of Zhang [22]. The column temperature was set to 35 °C. The mobile phases were (A) 0.5% (*v*/*v*) formic acid in water and (B) 0.5% (*v*/*v*) formic acid in acetonitrile [23]. The flow rate was set at 0.6 mL/min, and 5 μL of each sample was injected after equilibration. The elution program was used as follows: 0–1 min, 5% B; 1–8 min, 5–25% B; 8–12 min, 25–60% B; 12–13 min, 60–100% B; 13–16 min, 100% B; 16–16.1 min, 5% B; 16.1–20 min, 5% B. The UV detection was carried out at wavelengths of 260 nm and 254 nm. Phenolic compounds were identified by comparing retention times and UV spectra with authentic standards analyzed under identical chromatographic conditions. Quantification was based on external calibration curves constructed with these standards.

### 2.13. Volatile Compound

Volatiles were analyzed by GC-MS (Clarus SQ8, PerkinElmer, Waltham, MA, USA) according to the modified method of Manousi and Zachariadis [24]. An aliquot of 5 mL of edamame-based beverage was placed into a vial, followed by the addition of 50 mg of salt, which was mixed and dissolved thoroughly. The volatile compounds were extracted using a headspace sampling technique with a Headspace Model TM 110 Trap and analyzed by GC-MS. The headspace vial was heated at 80 °C for 10 min before introducing the volatiles into a GC-MS system. The oven temperature was initially set at 45 °C for 1 min and then increased at a rate of 7 °C per min until reaching a final temperature of 200 °C. Separation was carried out using an Elite-5ms column. The obtained mass spectra were analyzed using TurboMass software version 5.4 (PerkinElmer, Shelton, CT, USA) with the LibSearch Text library.

### 2.14. Statistical Analysis

Statistical analysis aimed to identify significant differences in sheet attributes. We employed analysis of variance (ANOVA) at a 95% confidence level for this purpose. To further discern specific variations between different mixes, Duncan’s multiple range test was utilized. All computations were carried out using SPSS 17 (SPSS Inc., Chicago, IL, USA).

## 3. Results and Discussion

### 3.1. Nutritional Composition

The proximate composition of the edamame-based beverage formulations varied significantly depending on the type and concentration of added components. Table 1 presents the nutritional profiles of six formulations prepared at two dilution ratios (1:3 and 1:6). The incorporation of coconut milk and coconut water, as well as the extent of dilution, significantly influenced the levels of protein, fat, carbohydrate, dietary fiber, ash, and total caloric content. Among all samples, the ECM (1:3) formulation exhibited the highest protein content at 3.68 g/100 g, likely due to the contribution of coconut milk, which contains approximately 3.28–3.60 g/100 g protein [2]. In contrast, the lowest protein content was observed in E (1:6) and ECW (1:6) samples (1.48 g/100 g), reflecting the dilution effect. Notably, edamame-based beverage alone still provided higher protein content (2.95 g/100 g) than many commercial plant-based beverages, such as almond (0.8–1.7 g/100 g), cashew (2.05 g/100 g), walnut (1.3–1.7 g/100 g), and oat beverage (0.78 g/100 g) [2]. Total dietary fiber ranged from 0.62 to 2.57 g/100 g. The highest value was found in ECW (1:3), likely due to the presence of suspended fibrous components from coconut water. Conversely, the lowest fiber content was noted in E (1:6), which is consistent with its high dilution and lower solid fraction. Interestingly, an edamame-based beverage at a 1:3 ratio also exceeded the fiber levels found in commercial cashew (1.15 g/100 g), oat (0.8 g/100 g), and soy beverages (0.70 g/100 g) [25]. Ash content, which is an indicator of total mineral content, was highest in the ECW (1:3) formulation (0.51 g/100 g), attributable to the mineral-rich profile of coconut water. The lowest ash value was seen in E (1:6) (0.17 g/100 g), consistent with reduced solids in diluted formulations.

Caloric content varied considerably across samples. ECM (1:3) exhibited the highest total energy (114.90 Kcal/100 g) and calories from fat (86.72 Kcal/100 g), followed closely by ECM (1:6) (114.06 and 94.82 Kcal/100 g, respectively), reflecting the lipid-rich nature of coconut milk. In contrast, E (1:6) provided the lowest energy values (18.89 Kcal/100 g total, 7.42 Kcal/100 g from fat). Carbohydrate content peaked in the ECW (1:3) sample (3.54 g/100 g), while fat content was highest in the ECM (1:6) (10.54 g/100 g), suggesting that both the type of coconut product and dilution level substantially modulate macronutrient delivery. As expected, moisture content was inversely related to concentration. The most diluted formulation, E (1:6), had the highest moisture content (96.14 g/100 g), whereas ECM (1:3) exhibited the lowest (82.85 g/100 g). These results confirm that higher solid content contributes to lower moisture levels and a more nutrient-dense product.

### 3.2. Mineral Composition

The mineral composition of the six edamame-based beverage formulations is summarized in Table 2. All samples contained similar elemental profiles, including magnesium (Mg), phosphorus (P), sulfur (S), chlorine (Cl), potassium (K), calcium (Ca), and zinc (Zn), though their concentrations varied depending on formulation. Calcium was observed in the highest relative concentrations across all samples, ranging from 19.25% to 27.64% of total mineral content. This is notable given that calcium is typically a limiting micronutrient in plant-based beverages and is often the focus of nutritional fortification [2]. The lowest concentrations were recorded for magnesium, chlorine, and zinc, which are nevertheless essential trace elements in human nutrition. Samples containing coconut water (ECW (1:3) and ECW (1:6)) exhibited elevated potassium levels, consistent with previous findings on coconut water’s high electrolyte content [18]. Interestingly, samples also showed differing sulfur levels. Two formulations—E (1:3) and E (1:6)—demonstrated relatively high sulfur concentrations (1.38–1.50%), whereas other formulations showed a broader sulfur range of 4.78–14.04%.

Based on the mineral distribution, the samples can be categorized into three compositional groups namely (i) ECW (1:3) and ECW (1:6), characterized by elevated levels of potassium, calcium, and phosphorus; (ii) E (1:3) and E (1:6), displaying high levels of sulfur and magnesium; and (iii) ECM (1:3) and ECM (1:6), which contained consistently high potassium and moderate amounts of calcium, phosphorus, and magnesium. These variations reflect the mineral contribution of coconut-derived ingredients and dilution ratios. The grouping also suggests possible applications for targeted nutritional enhancement, such as developing formulations optimized for electrolyte replacement or bone health support.

### 3.3. Amino Acid Profile and Concentrations

The qualitative and quantitative profiles of amino acids in the six edamame-based beverage formulations are presented in Table 3. Amino acids were classified into hydrophilic (polar), hydrophobic (nonpolar), and neutral categories [26]. Across all samples, glutamic acid and aspartic acid were the most abundant amino acids, consistent with previous reports for plant-based beverages such as oat and almond milk [27]. In particular, the E (1:3) sample exhibited the highest total concentration of amino acids across all categories, suggesting a nutritionally denser formulation, potentially due to higher solid content or reduced dilution effects. Among hydrophilic amino acids, glutamic acid and aspartic acid were predominant, with peak concentrations of 5.08 mg/mL and 3.06 mg/mL, respectively, observed in the E (1:3) sample. These amino acids possess negatively charged carboxylate side chains (COO^−^) that contribute to umami flavor and buffering capacity, both of which are important for the sensory and physicochemical properties of plant-based beverages. Lysine, alanine, and arginine were also detected in appreciable amounts, contributing to the protein quality of the beverage. In particular, lysine is an essential amino acid often limited in cereal-based beverages, enhancing the value of edamame as a beverage.

Hydrophobic amino acids such as alanine, valine, leucine, and phenylalanine were more concentrated in the E (1:3) formulation. These nonpolar amino acids play key roles in protein folding and hydrophobic interactions, potentially influencing the structural integrity, texture, and emulsion stability of the beverage. The presence of branched-chain amino acids (BCAAs), particularly leucine and isoleucine, further enhances the nutritional profile due to their roles in muscle protein synthesis and metabolic regulation. Neutral amino acids, including serine, glycine, threonine, and proline, were present in moderate concentrations across all samples, again with the highest levels observed in E (1:3). These amino acids contribute to protein solubility, hydrogen bonding, and structural flexibility, potentially affecting the emulsifying and functional behavior of the beverage matrix. Overall, the consistent predominance of glutamic acid underscores its central role in defining both the functional and organoleptic attributes of edamame-based beverages. The relatively higher concentrations of both polar and nonpolar amino acids in the E (1:3) formulation suggest that this sample provides superior nutritional density and may offer enhanced functional performance in terms of flavor, stability, and texture.

### 3.4. Total Phenolics, Total Flavonoids, and Antioxidant Activity

The total phenolic content (TPC), total flavonoid content (TFC), and DPPH radical scavenging activity of edamame-based beverage and its formulations are summarized in Table 4. Plant-based beverages derived from nuts and legumes, including edamame, are known to contain bioactive compounds with antioxidant properties that can mitigate the risk of chronic diseases such as cardiovascular disorders, diabetes, and cancer by inhibiting oxidative damage to lipids, proteins, and nucleic acids [2]. Among all formulations, the E (1:3) sample exhibited significantly higher (*p* ≤ 0.05) TPC and TFC than the E (1:6) formulation, indicating that higher concentrations of edamame solids contribute to enhanced antioxidant capacity. This was reflected in the DPPH radical scavenging activity of E (1:3), which reached 14.85 ± 0.72%, nearly double that of E (1:6) (7.12 ± 0.65%).

The addition of coconut water or coconut milk at the 1:3 ratio appeared to reduce TPC and DPPH activity in ECW (1:3) and ECM (1:3) compared to the E (1:3) control. This may result from a dilution effect or interactions between phenolic compounds and components introduced by the coconut matrices, potentially affecting compound extractability or stability. Interestingly, in the 1:6 dilution group, the inclusion of coconut water or coconut milk led to enhanced antioxidant activity, with DPPH values of 10.32 ± 0.62% for ECW (1:6) and 9.95 ± 0.67% for ECM (1:6), both significantly higher than E (1:6) (*p* ≤ 0.05). These findings suggest that coconut-based components may contribute synergistic or complementary antioxidant effects under diluted conditions.

As measured by the DPPH assay, antioxidant activity reflects the capacity of compounds to donate electrons or hydrogen atoms to stabilize free radicals [7]. Regarding flavonoid content, ECW (1:3) demonstrated a marked reduction in TFC (3.48 ± 0.66 mg QE/100 mL) compared to E (1:3) (6.42 ± 0.53 mg QE/100 mL), a nearly 50% decrease. This effect, however, was less pronounced at the 1:6 dilution level. Notably, the addition of coconut milk had no significant effect on TFC in either concentration group, suggesting that its impact on antioxidant phytochemicals may be limited. Among all tested formulations, ECM (1:3) provided a favorable balance by retaining relatively high levels of phenolic and flavonoid compounds while maintaining substantial DPPH scavenging activity. These findings highlight the potential of edamame-based beverages, even when blended with other plant-based ingredients, to serve as a functional beverage with retained antioxidant activity.

### 3.5. Particle Size Distribution

Plant-based beverages are complex colloidal dispersions comprising diverse constituents such as fat globules, proteins, polysaccharides, and insoluble plant particles. These components vary in both size and shape and collectively contribute to the physical and sensory properties of the final product. However, such structural complexity often results in instability during storage, manifested by sedimentation, creaming, or phase separation [1]. Particle size and its distribution are therefore critical indicators of product stability and quality. The particle size distribution of edamame-based beverage at different concentrations (E (1:3) and E (1:6)) and its formulations with coconut water (ECW) and coconut milk (ECM) is summarized in Table 5. Four size parameters were used to characterize the dispersions: D10 (10th percentile), D50 (median diameter), D90 (90th percentile), and D_av_ (volume-weighted mean diameter).

The E (1:6) sample exhibited values of D10 = 9.36 ± 0.06 µm, D50 = 54.17 ± 0.23 µm, D90 = 140.33 ± 0.58 µm, and Dav = 65.80 ± 0.17 µm, which were smaller than those observed for the E (1:3) sample (D10 = 8.31 ± 0.08 µm, D50 = 57.90 ± 0.26 µm, D90 = 151.00 ± 0.00 µm, and Dav = 70.27 ± 0.06 µm). This indicates a broader and larger particle size distribution in the more concentrated formulation, likely due to a higher proportion of insoluble solids and reduced dilution.

**Figure 1:**
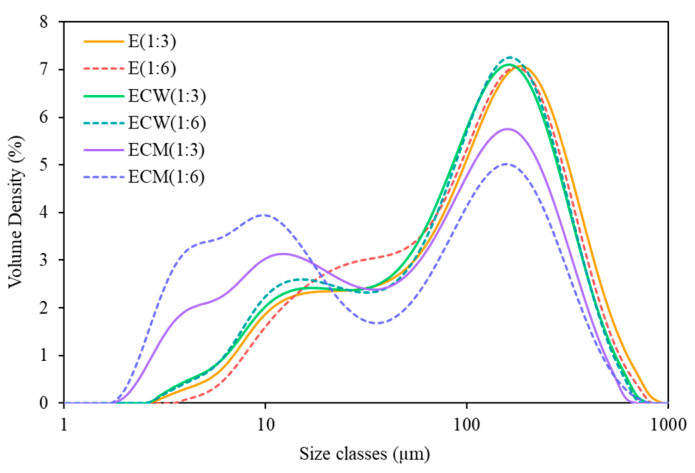
Particle size distribution curves of edamame-based beverage (E) and its blends with coconut milk (ECM) and coconut water (ECW) at ratios of 1:3 and 1:6, measured using a laser diffraction particle size analyzer.

The addition of coconut water and coconut milk significantly decreased the particle size compared to both E (1:3) and E (1:6) samples. Notably, the ECM (1:6) sample showed the smallest particle sizes among all formulations, with values of D10 = 2.86 ± 0.04 µm, D50 = 22.20 ± 0.46 µm, D90 = 113.33 ± 2.52 µm, and Dav = 43.53 ± 1.20 µm. The improved size distribution is likely due to the emulsifying properties of coconut milk, which contains natural surface-active proteins and lipids that enhance emulsion stability and limit aggregation [28]. Figure 1 visually supports these findings, showing that formulations containing coconut milk achieved narrower particle size distributions compared to those with coconut water. The smaller and more uniform particle size observed in ECM samples is critical for improving product stability, mouthfeel, and overall sensory acceptance in plant-based beverages.

### 3.6. Rheological Behavior

**Figure 2:**
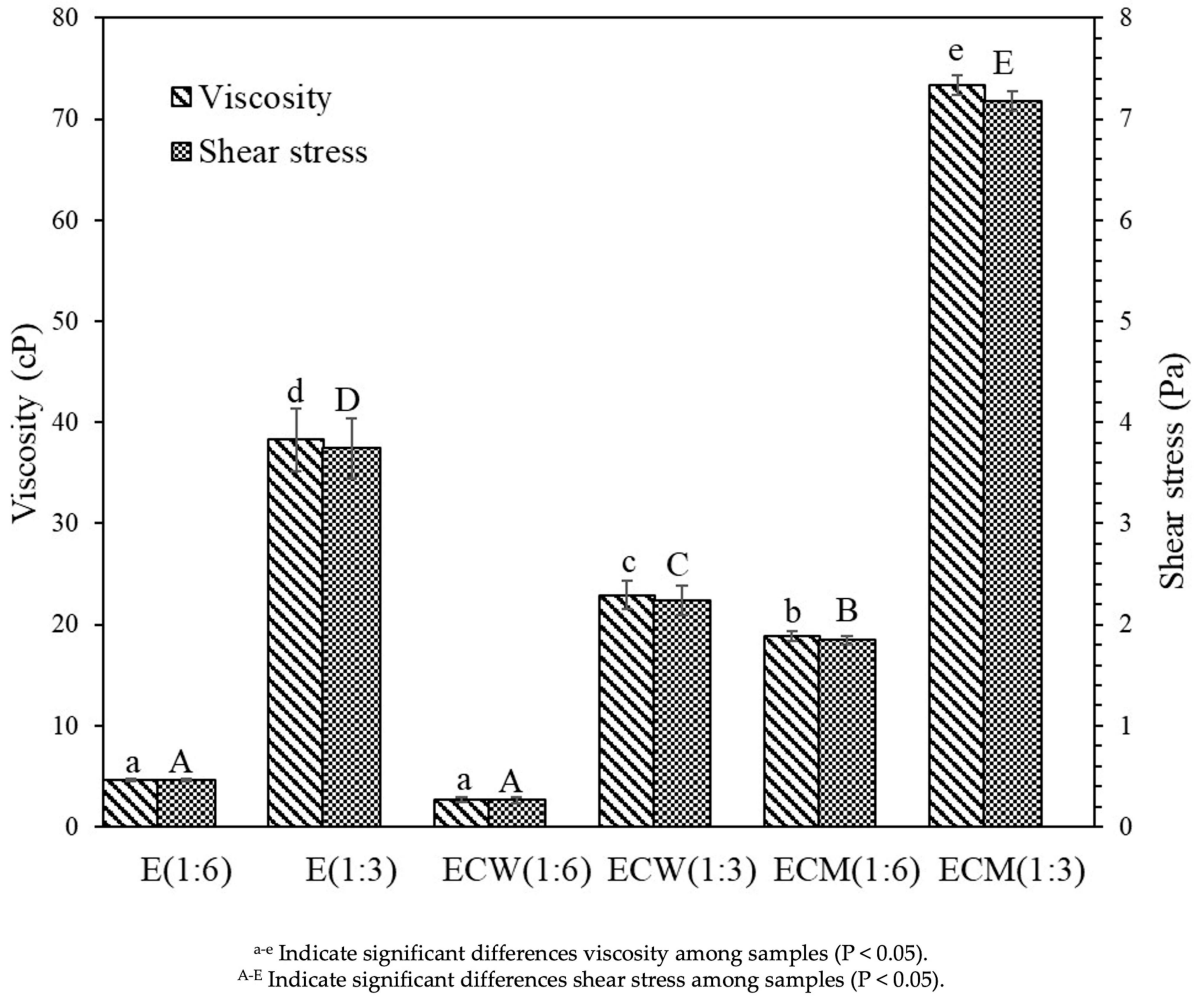
Viscosity (cP) and shear stress (Pa) of edamame-based beverage (E), ECW, and ECM formulations at different dilution ratios (1:3 and 1:6), measured at room temperature.

The rheological characteristics of edamame-based beverage and its formulations with coconut milk (CM) or coconut water (CW) were assessed by measuring viscosity (cP) and shear stress (dyne/cm^2^), as illustrated in Figure 2. These parameters provide critical insight into the flow behavior, textural quality, and processability of colloidal beverages. Among all samples, ECM (1:3) exhibited the highest viscosity and shear stress values, indicating the formation of a denser and more cohesive matrix with increased resistance to deformation under applied force. This rheological enhancement can be attributed to the higher fat and protein content in the ECM formulation (Table 1), which likely promoted stronger intermolecular interactions, resulting in a thicker and more stable emulsion. Conversely, the ECW (1:6) sample showed the lowest values for both viscosity and shear stress, reflecting a more fluid, less structured system with reduced resistance to flow. This behavior is consistent with its higher water content, lower solids concentration, and absence of fat-based structuring agents.

The observed differences in rheological properties across formulations are primarily influenced by compositional factors (e.g., fat and protein content, emulsifier presence) and processing conditions (e.g., dilution ratio, blending). Notably, the incorporation of coconut milk, which naturally contains emulsifying agents such as phospholipids and proteins, may have enhanced colloidal stability by promoting emulsification and reducing phase separation. A strong positive correlation was observed between viscosity and shear stress across all samples, suggesting that both parameters are governed by similar physicochemical determinants, including particle size distribution, emulsifier concentration, and total solids content. The ability of the formulations to maintain consistent rheological profiles is crucial for ensuring consumer acceptability, as well as industrial performance during processing, filling, transportation, and storage. These findings demonstrate that formulation strategy, particularly the type and concentration of plant-based ingredients, plays a central role in modulating the flow behavior of plant-based beverage systems, which in turn affects mouthfeel, stability, and shelf-life.

### 3.7. Separation Index

**Figure 3:**
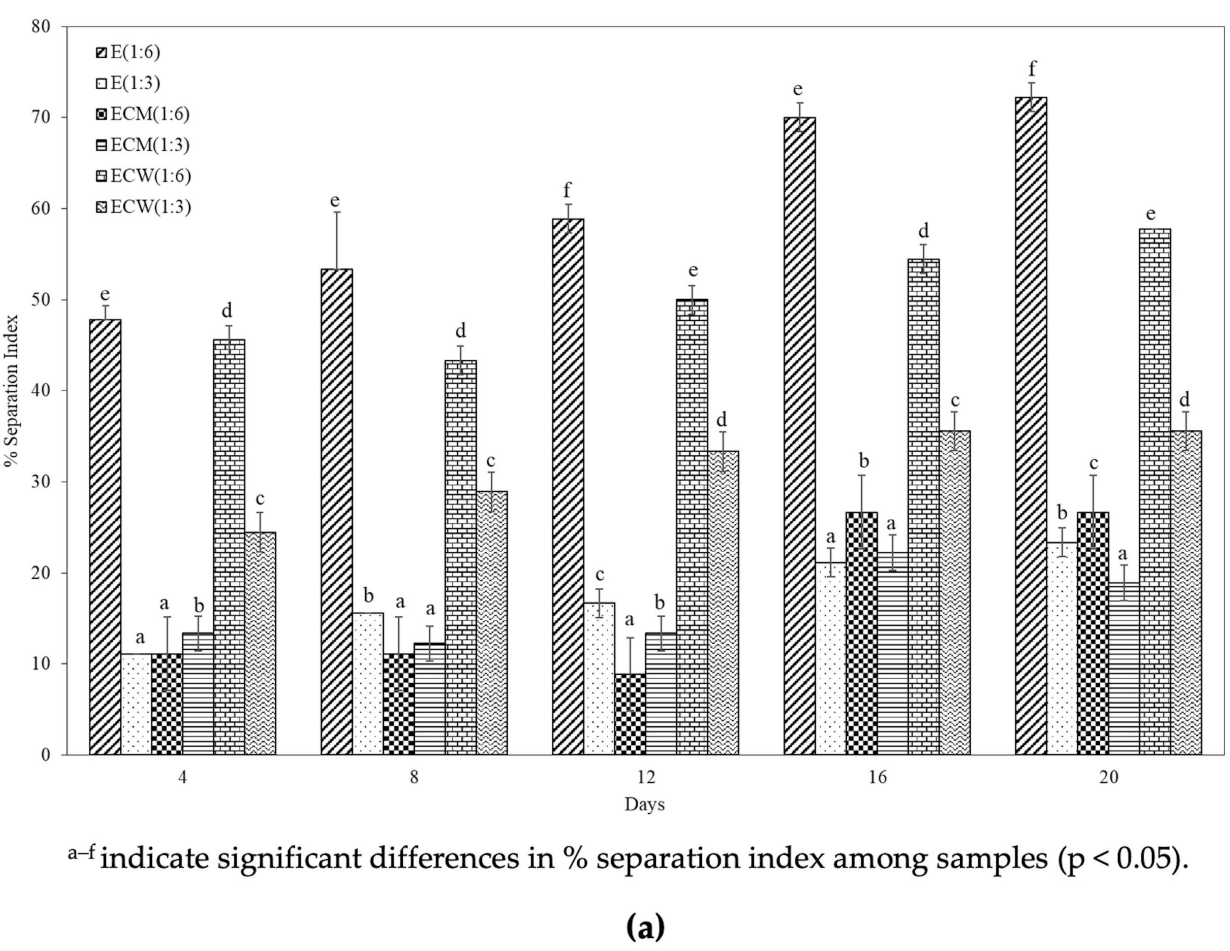

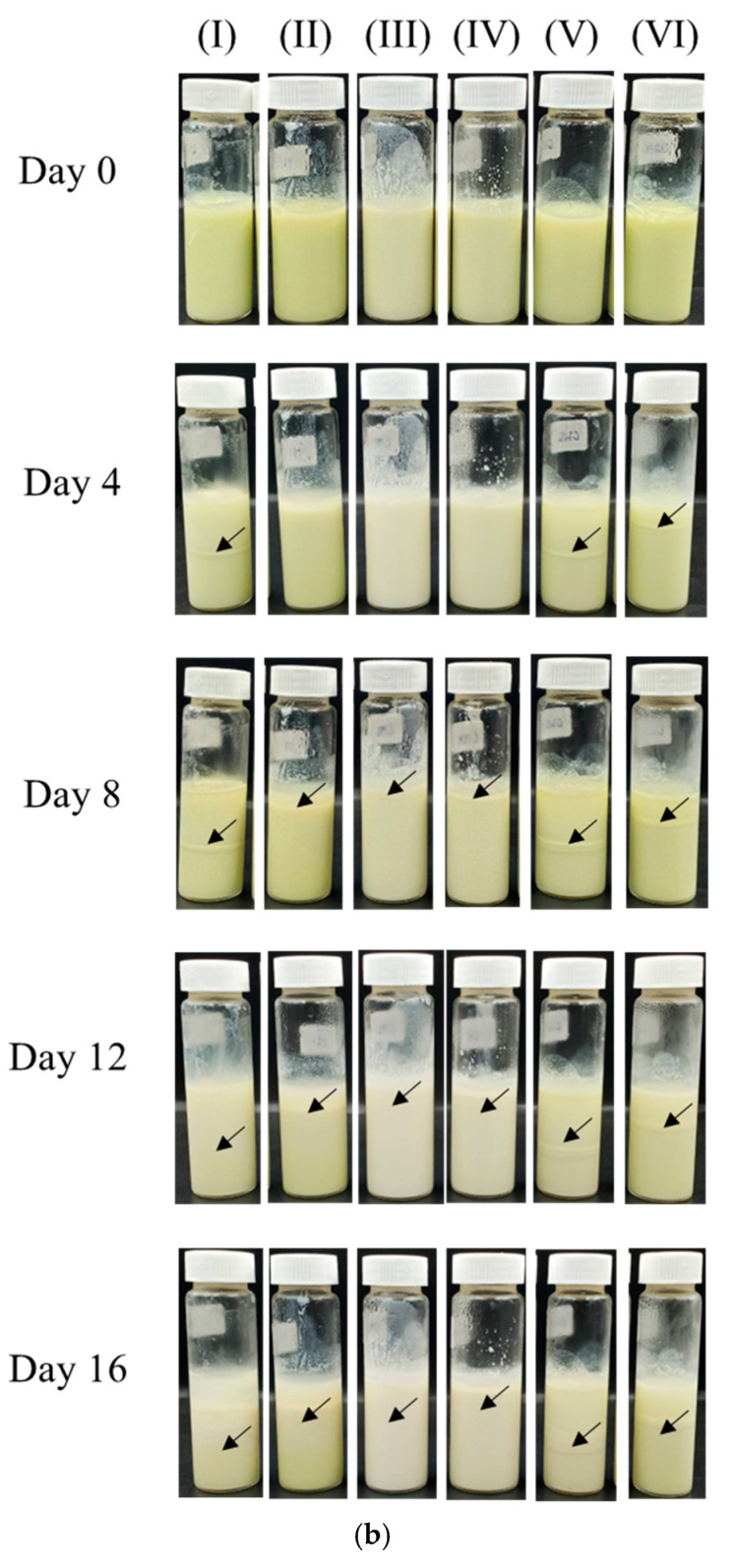
(**a**) Separation index (%) and (**b**) Physical appearance of edamame-based beverage formulations during storage at 4 °C for 20 days: (I) E (1:6), (II) E (1:3), (III) ECM (1:6), (IV) ECM (1:3), (V) ECW (1:6), and (VI) ECW (1:3). Photographs were taken at the end of the storage period.

The physical stability of plant-based beverage systems is closely influenced by the size and interaction of dispersed particles such as fat globules, proteins, and insoluble solids, which can lead to sedimentation and phase separation during storage. The separation index (SI), expressed as a percentage, was used to quantify this stability in various edamame-based beverage formulations over a 20-day storage period. Figure 3 illustrates SI trends in six formulations: E (1:6), E (1:3), ECW (1:6), ECW (1:3), ECM (1:6), and ECM (1:3), measured on Days 4, 8, 12, 16, and 20. The E (1:6) formulation consistently exhibited the highest SI values throughout the storage period, indicating greater phase separation. This is likely due to its lower solids content, which reduces the viscosity and weakens interparticle interactions, facilitating sedimentation. In contrast, the E (1:3) formulation demonstrated significantly lower SI values at all time points, consistent with its higher content of suspended solids and protein, which contributed to enhanced colloidal stability.

Among the formulations enriched with coconut milk, ECM (1:3) exhibited the lowest SI values during the early stages of storage (up to Day 12), highlighting superior short-term stability. However, a modest increase in separation was observed on Days 16 and 20. This delayed separation may result from structural rearrangement, coalescence, or delayed emulsification dynamics as the coconut milk components interact with the protein-rich matrix over time. The improved stability of ECM formulations is attributed to the presence of natural emulsifying agents in coconut milk, particularly coconut proteins, which can absorb oil droplets and facilitate the formation of stable oil-in-water (O/W) emulsions [28,29]. This mechanism is supported by prior findings from Table 3, which indicated abundant hydrophilic and hydrophobic amino acids in the E (1:3) and ECM formulations, and by Table 5, which showed significantly reduced particle sizes in ECM (1:3) and ECM (1:6), further promoting dispersion stability. In comparison, ECW (1:6) demonstrated slightly lower SI values than E (1:6) and exhibited steady improvement in stability over time. This may be due to the high-water content enhancing solubility and mobility of coconut water-soluble solids, while contributing less to viscosity than coconut milk. The ECW (1:3) sample showed intermediate separation, with a gradual upward trend across the storage period. These findings underscore the importance of formulation ratio and type of aqueous phase (coconut milk vs. coconut water) in influencing the colloidal and phase stability of plant-based beverage systems. Lower separation indexes, particularly in ECM (1:3), are desirable attributes for ensuring uniform product appearance, sensory acceptability, and shelf-life stability. Moreover, the results suggest that optimizing the fat content, protein concentration, and aqueous phase composition can be a strategic approach to enhance the long-term stability of functional plant-based beverages.

### 3.8. Phenolic Compounds Profile

**Figure 4:**
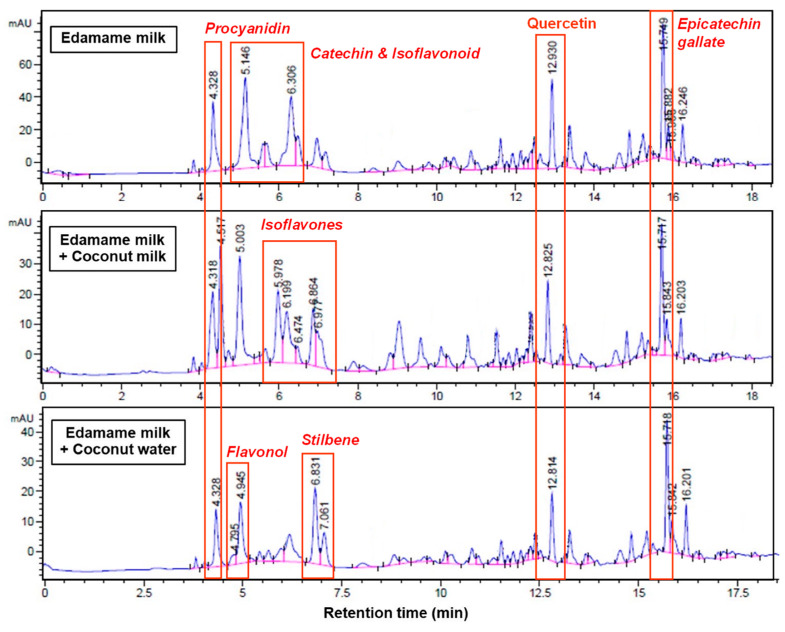
HPLC chromatograms (260 nm) showing phenolic compound profiles in edamame-based beverage (E), and its blends with coconut milk (ECM) and coconut water (ECW). Peaks indicate key polyphenols identified in each formulation.

Edamame is a rich source of isoflavones, a subclass of polyphenolic compounds belonging to the flavonoid family with known antioxidant and phytoestrogenic properties [30]. Analysis of the phenolic profile of edamame-based beverages and their formulations revealed distinct chromatographic patterns. As shown in the HPLC chromatograms (Figure 4), three early retention time peaks were consistently observed in plain edamame-based beverage: procyanidin B-dimer (4.33 min), catechin/epicatechin (5.15 min), and coumestrol (6.31 min)—compounds typically associated with the antioxidant profile of soy-based products [31]. All formulations also exhibited two late peaks, identified as quercetin (~12.83–12.93 min) and (–)-epicatechin gallate (~15.7 min), both of which are commonly reported in legume-based and polyphenol-rich matrices. The incorporation of coconut milk introduced four new phenolic peaks at retention times of 4.52, 5.00, 5.98, and 6.86 min, corresponding to apigenin glycoside, acetyl- and malonyl-isoflavones, and formononetin, respectively [22]. These coconut-derived flavonoids may have contributed to the shift in phenolic profile, with the observed reduction in catechin signal suggesting possible competitive binding, matrix interaction, or transformation during processing.

Among the newly detected compounds, apigenin glycoside is particularly notable. It is a naturally occurring flavonoid reported in coconut milk with potential anti-inflammatory, antioxidant, and anticancer properties [32]. However, the altered profile in ECM (1:3), particularly the diminished catechin peak, is consistent with its reduced DPPH radical scavenging activity, as previously discussed in Section 3.4. In contrast, the addition of coconut water enhanced the phenolic diversity without depleting the native edamame antioxidants. Specifically, quercetin–glucoside (isoquercitrin, RT = 4.95 min) and polydatin (RT = 6.83 min) were newly identified in ECW (1:6). These compounds are known to exist in coconut water [33] and other plant sources such as grapes and peanuts [34]. Quercetin–glucoside, a flavonol, and polydatin, a stilbene glucoside, both exhibit strong antioxidant activity, which likely contributed to the higher DPPH value observed in ECW (1:6) compared to E (1:6).

### 3.9. Volatile Compound Profile

**Figure 5:**
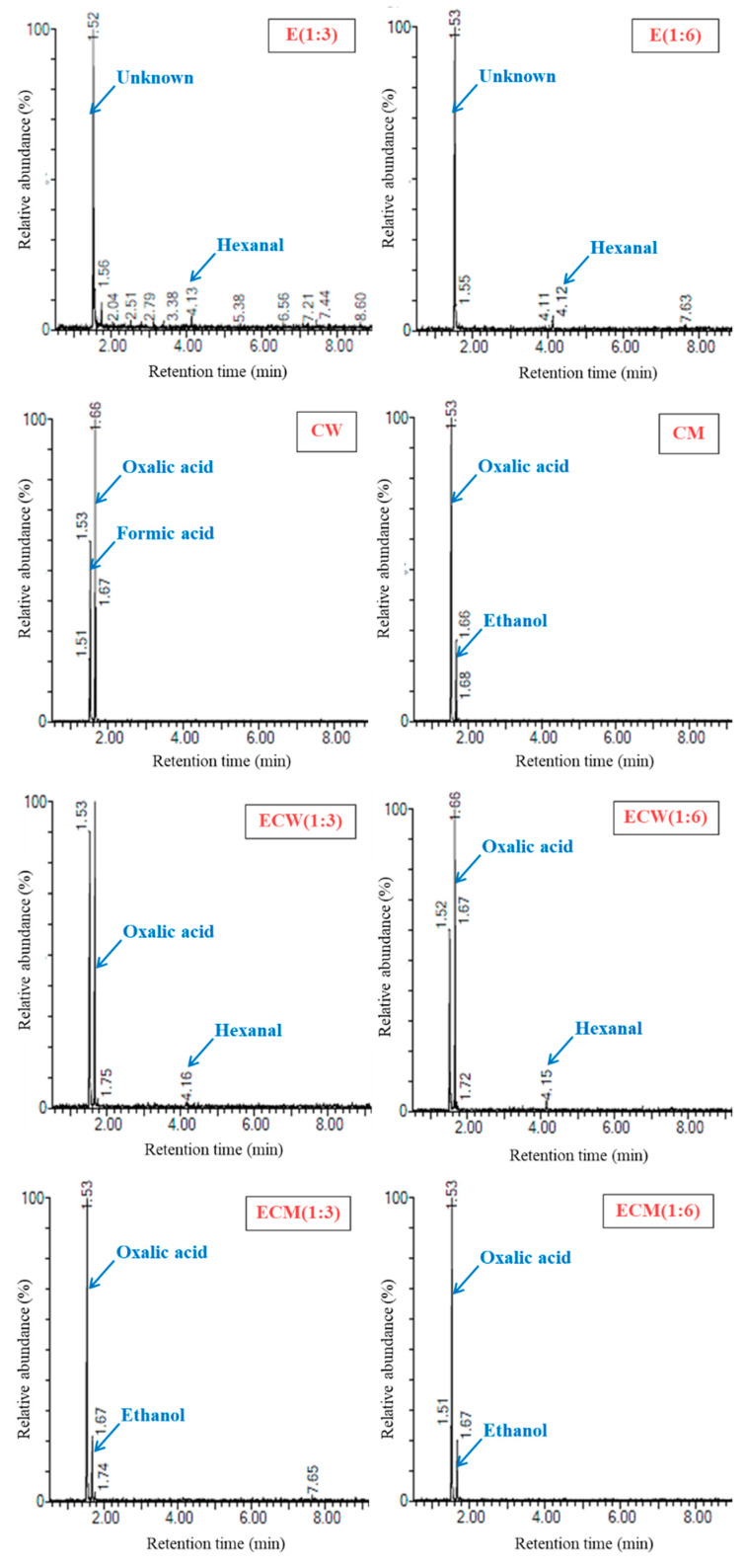
GC–MS chromatograms illustrating the volatile compound profiles of edamame-based beverage (E) and its formulations with coconut water (ECW) and coconut milk (ECM). Key aroma-active volatiles are annotated.

Figure 5 presents the gas chromatography–mass spectrometry (GC–MS) profiles of edamame-based beverage, coconut water, coconut milk, and their respective mixed formulations. Volatile compound analysis provides valuable insight into the sensory characteristics and acceptability of plant-based beverage products, particularly in masking undesirable off-flavors. The volatile profile of the edamame-based beverage was dominated by hexanal, a major aldehyde known to impart green, grassy, or raw-bean odors, commonly perceived as beany off-flavor in legume-based beverages [8]. Additional volatiles such as 1-octen-3-ol and octanal were also present and are known for their high odor activity values (OAVs) in soybean matrices [16], further contributing to the overall beany aroma. In contrast, coconut water exhibited formic acid and oxalic acid as its major volatiles. These organic acids are associated with mildly sweet and sour flavor notes, contributing to the light and refreshing sensory character of coconut water [35]. In coconut milk, oxalic acid was also detected alongside ethanol, which plays a role in the generation of volatile esters through esterification reactions with fatty acids. These esters can impart alcoholic, sweet, or fruity aromas, enhancing the sensory appeal [15,16].

The incorporation of coconut derivatives significantly altered the volatile profile of the edamame-based beverage. Formulations with coconut milk (ECM (1:3) and ECM (1:6)) showed a marked reduction in hexanal intensity and a corresponding shift in the volatile fingerprint toward compounds characteristic of coconut milk. This suggests a more effective masking of the beany odor by coconut milk compared to coconut water. The ECW (1:3) and ECW (1:6) samples retained noticeable levels of hexanal, indicating incomplete masking and the persistence of legume-derived odors. These observations highlight the superior sensory-modifying capacity of coconut milk, likely due to its lipid-rich matrix, which promotes partitioning or dilution of volatiles and introduces complementary aroma compounds, reducing undesirable odors and enhancing sensory quality. Furthermore, its natural emulsifying agents may facilitate molecular interactions that suppress volatile aldehydes such as hexanal.

## 4. Conclusions

This study demonstrates the potential of edamame-based beverages as a nutritionally dense and functionally stable plant-based beverage. Formulations with a higher edamame-to-water ratio (1:3) exhibited improved colloidal stability, supporting suitability for extended storage and a longer shelf life. The 1:3 formulation also showed greater nutritional density than E (1:6), evidenced by higher concentrations of key amino acids (e.g., glutamic acid, aspartic acid, leucine) and antioxidant constituents (total phenolics and flavonoids). Incorporating coconut milk significantly enhanced emulsification, reduced particle size, and improved rheological properties, thereby increasing physical stability and mouthfeel. By contrast, coconut water supplied additional bioactive compounds (e.g., quercetin-glucoside and polydatin), augmenting the antioxidant profile without compromising colloidal stability. Overall, blending edamame with coconut-derived components offers a promising strategy for developing dairy-free beverages with improved sensory appeal, nutritional functionality, and consumer acceptability, particularly for those seeking high-protein or functional-food options. Moreover, further in vivo and clinical studies should be conducted to substantiate these benefits for diabetics/CKD patients.

## Figures and Tables

**Table 1:** Nutritional composition of edamame-based beverage (E) and edamame-based beverage blended with coconut milk (ECM) or coconut water (ECW) at dilution ratios of 1:3 and 1:6.

	E (1:3)	ECM (1:3)	ECW (1:3)	E (1:6)	ECM (1:6)	ECW (1:6)
**Ash (g/100 g)**	0.28 ± 0.00 ^b^	0.48 ± 0.01 ^e^	0.51 ± 0.01 ^f^	0.17 ± 0.00 ^a^	0.36 ± 0.01 ^c^	0.39 ± 0.01 ^d^
**Calories (Kcal/100 g)**	36.09 ± 0.04 ^c^	114.90 ± 0.15 ^f^	42.46 ± 0.40 ^d^	18.89 ± 0.03 ^a^	114.06 ± 0.49 ^e^	29.50 ± 0.23 ^b^
**Calories from fat (Kcal/100 g)**	15.39 ± 0.18 ^c^	86.72 ± 0.41 ^e^	19.92 ± 0.18 ^d^	7.42 ± 0.05 ^a^	94.82 ± 0.77 ^f^	9.86 ± 0.14 ^b^
**Carbohydrates (g/100 g)**	2.23 ± 0.03 ^c^	3.37 ± 0.05 ^d^	3.54 ± 0.19 ^d^	1.39 ± 0.02 ^a^	1.97 ± 0.05 ^b^	3.44 ± 0.11 ^d^
**Fat (g/100 g)**	1.71 ± 0.02 ^c^	9.64 ± 0.05 ^e^	1.88 ± 0.02 ^d^	0.83 ± 0.01 ^a^	10.54 ± 0.09 ^f^	1.10 ± 0.02 ^b^
**Moisture (g/100 g)**	92.84 ± 0.02 ^d^	82.85 ± 0.02 ^a^	91.23 ± 0.12 ^c^	96.14 ± 0.00 ^f^	84.30 ± 0.02 ^b^	93.61 ± 0.07 ^e^
**Protein (g/100 g)**	2.95 ± 0.01 ^c^	3.68 ± 0.01 ^d^	2.85 ± 0.03 ^b^	1.48 ± 0.01 ^a^	2.84 ± 0.02 ^b^	1.48 ± 0.01 ^a^
**Total dietary fiber (g/100 g)**	1.86 ± 0.00 ^c^	2.43 ± 0.01 ^e^	2.57 ± 0.01 ^f^	0.62 ± 0.00 ^a^	1.89 ± 0.01 ^d^	1.09 ± 0.00 ^b^

Data are expressed as mean ± SD. Different letters in the same row indicate significant differences (*p* < 0.05).

**Table 2:** Mineral composition of edamame-based beverage formulations: E (pure edamame-based beverage), ECM (edamame + coconut milk), and ECW (edamame + coconut water) at dilution ratios of 1:3 and 1:6.

Element	Mass Concentration (Mass%)					
	E (1:3)	ECM (1:3)	ECW (1:3)	E (1:6)	ECM (1:6)	ECW (1:6)
**Mg**	3.09 ± 0.10 ^bc^	2.87 ± 0.23 ^b^	2.21 ± 0.23 ^a^	3.42 ± 0.40 ^cd^	3.64 ± 0.12 ^d^	2.05 ± 0.26 ^a^
**P**	13.38 ± 0.11 ^c^	10.55 ± 0.60 ^b^	8.37 ± 0.15 ^a^	15.65 ± 0.86 ^d^	10.75 ± 0.18 ^b^	8.68 ± 0.15 ^a^
**S**	15.26 ± 0.42 ^c^	12.45 ± 2.41 ^b^	9.79 ± 0.70 ^a^	20.00 ± 2.40 ^d^	15.19 ± 0.81 ^c^	9.91 ± 0.71 ^a^
**Cl**	1.38 ± 0.40 ^a^	4.78 ± 0.60 ^b^	10.75 ± 0.31 ^d^	1.50 ± 0.21 ^a^	7.04 ± 1.47 ^c^	14.04 ± 0.48 ^e^
**K**	38.41 ± 0.50 ^b^	44.04 ± 1.84 ^c^	44.79 ± 0.94 ^c^	34.96 ± 2.52 ^a^	40.82 ± 0.19 ^b^	44.67 ± 0.61 ^c^
**Ca**	27.64 ± 0.28 ^d^	24.67 ± 2.18 ^c^	22.72 ± 0.86 ^b^	22.66 ± 0.35 ^b^	21.51 ± 0.87 ^b^	19.25 ± 0.56 ^a^
**Zn**	0.22 ± 0.04 ^b^	0.31 ± 0.02 ^c^	0.12 ± 0.04 ^a^	0.12 ± 0.02 ^a^	0.28 ± 0.08 ^bc^	0.12 ± 0.02 ^a^

Data are expressed as mean ± SD. Different letters in the same row indicate significant differences (*p* < 0.05).

**Table 3:** Amino acid profiles and total concentrations of edamame-based beverage (E), coconut water blends (ECW), and coconut milk blends (ECM) at dilution ratios of 1:3 and 1:6.

Type	Amino Acid (mg/mL)					
	E (1:3)	ECM (1:3)	ECW (1:3)	E (1:6)	ECM (1:6)	ECW (1:6)
**Hydrophilic**	**11.68**	**11.97**	**10.10**	**6.90**	**10.97**	**6.65**
Aspartic	3.06	2.83	2.65	1.81	2.49	1.75
Glutamic	5.08	5.25	4.42	2.96	4.78	2.88
Lysine *	1.80	1.68	1.51	1.06	1.40	1.05
Arginine	1.74	2.21	1.52	1.07	2.30	0.97
**Hydrophobic**	**6.98**	**6.66**	**6.08**	**4.05**	**6.32**	**3.84**
Alanine	1.12	1.17	0.98	0.65	1.07	0.62
Valine *	1.03	1.03	0.84	0.56	1.00	0.50
Cystine	0.07	0.05	0.05	0.03	0.07	0.01
Methionine *	0.27	0.29	0.28	0.18	0.29	0.19
Phenylalanine *	1.36	1.27	1.23	0.83	1.22	0.80
Isoleucine *	1.15	1.03	0.99	0.67	0.96	0.63
Leucine *	1.98	1.82	1.71	1.13	1.71	1.09
**Neutral**	**6.19**	**5.82**	**5.31**	**3.49**	**5.28**	**3.22**
Serine	1.59	1.54	1.43	0.93	1.41	0.88
Histidine *	0.67	0.63	0.60	0.40	0.55	0.38
Glycine	1.06	1.10	0.95	0.63	1.03	0.60
Threonine *	0.89	0.83	0.78	0.50	0.76	0.46
Tyrosine	0.76	0.60	0.51	0.36	0.54	0.22
Proline	1.22	1.12	1.04	0.67	0.99	0.68
**Total**	**24.85**	**24.45**	**21.49**	**14.44**	**22.57**	**13.71**

Data are expressed in mg/mL, where * indicates essential amino acids.

**Table 4:** Total phenolic content, total flavonoid content, and DPPH radical scavenging activity of edamame-based beverage formulations (E, ECW, and ECM) at dilution ratios of 1:3 and 1:6.

Sample	Total Phenolics (mg GAE/100 mL)	Total Flavonoids (mg QE/100 mL)	DPPH Scavenging (%)
**E (1:3)**	16.25 ± 0.39 ^c^	6.42 ± 0.53 ^c^	14.85 ± 0.72 ^d^
**E (1:6)**	9.45 ± 0.38 ^a^	5.10 ± 0.57 ^b^	7.12 ± 0.65 ^a^
**ECW (1:3)**	10.42 ± 0.43 ^b^	3.48 ± 0.66 ^a^	11.08 ± 2.20 ^bc^
**ECW (1:6)**	8.94 ± 0.22 ^a^	3.15 ± 0.27 ^a^	10.32 ± 0.62 ^b^
**ECM (1:3)**	10.50 ± 0.91 ^b^	6.10 ± 0.40 ^c^	12.33 ± 0.47 ^c^
**ECM (1:6)**	9.20 ± 0.82 ^a^	5.18 ± 0.40 ^b^	9.95 ± 0.67 ^b^

GAE refers to gallic acid equivalent. QE refers to quercetin equivalent. Data are expressed as mean ± SD. Different letters in the same column indicate significant differences (*p* < 0.05).

**Table 5:** Particle size characteristics (D10, D50, D90, and D_av_) of edamame-based beverage (E), edamame–coconut water (ECW), and edamame–coconut milk (ECM) formulations at dilution ratios of 1:3 and 1:6, determined via laser light scattering.

Samples	Particle Size (μm)			
	D_av_	D (10)	D (50)	D (90)
**E (1:3)**	70.27 ± 0.06 ^f^	8.31 ± 0.08 ^e^	57.90 ± 0.26 ^e^	151.00 ± 0.00 ^e^
**E (1:6)**	65.80 ± 0.17 ^e^	9.36 ± 0.06 ^f^	54.17 ± 0.23 ^d^	140.33 ± 0.58 ^d^
**ECW (1:3)**	62.83 ± 0.49 ^d^	7.43 ± 0.04 ^d^	52.00 ± 0.36 ^c^	135.00 ± 1.73 ^c^
**ECW (1:6)**	61.80 ± 0.30 ^c^	7.23 ± 0.05 ^c^	51.97 ± 0.31 ^c^	132.67 ± 0.58 ^c^
**ECM (1:3)**	50.07 ± 0.42 ^b^	3.85 ± 0.04 ^b^	35.63 ± 0.45 ^b^	121.00 ± 1.00 ^b^
**ECM (1:6)**	43.53 ± 1.20 ^a^	2.86 ± 0.04 ^a^	22.20 ± 0.46 ^a^	113.33 ± 2.52 ^a^

Data are expressed as mean ± SD. Different letters in the same column indicate significant differences (*p* < 0.05).

## Data Availability

The data presented in this study are available upon request from the corresponding author.
